# Perioperative Complications and Long-Term Follow-Up of Liver Transplantation in Hemorrhagic Hereditary Telangiectasia: Report of Three Cases and Systematic Review

**DOI:** 10.3390/jcm11195624

**Published:** 2022-09-24

**Authors:** Antoni Riera-Mestre, Pau Cerdà, Yoelimar Carolina Guzmán, Adriana Iriarte, Alba Torroella, José María Mora-Luján, Jose Castellote, Amelia Hessheimer, Constantino Fondevila, Laura Lladó

**Affiliations:** 1 Hemorrhagic Hereditary Telangiectasia Unit, Hospital Universitari Bellvitge, L’Hospitalet de Llobregat, 08907 Barcelona, Spain; 2 Internal Medicine Department, Hospital Universitari Bellvitge, L’Hospitalet de Llobregat, 08907 Barcelona, Spain; 3 Bellvitge Biomedical Research Institute (IDIBELL), L’Hospitalet de Llobregat, 08907 Barcelona, Spain; 4 Faculty of Medicine and Health Sciences, Universitat de Barcelona, 08007 Barcelona, Spain; 5 General & Digestive Surgery, Institut Clínic de Malalties Digestives i Metabòliques, Hospital Clínic, 08036 Barcelona, Spain; 6 Liver Transplant Unit, Gastroenterology Department, Hospital Universitari Bellvitge, 08907 Barcelona, Spain; 7 Hepatopancreatobiliary Surgery & Transplantation, General & Digestive Surgery, Hospital Universitario La Paz, IdiPAZ, CIBERehd, 28046 Madrid, Spain; 8 Liver Transplant Unit, Surgery Department, Hospital Universitari Bellvitge, 08907 Barcelona, Spain

**Keywords:** hemorrhagic hereditary telangiectasia, vascular malformation, liver transplantation, angiogenesis

## Abstract

The aim was to describe three patients with hemorrhagic hereditary telangiectasia (HHT) requiring liver transplantation (LT) and to perform a systematic review focusing on surgical complications and long-term follow-up. Unrestricted searches of the Medline and Embase databases were performed through February 2022. Forty-five studies were selected including 80 patients plus the three new reported patients, 68 (81.9%) were female and mean age was 50 (27–72) years. Main indications for LT were high-output cardiac failure (*n* = 40; 48.2%), ischemic cholangitis (*n* = 19; 22.9%), and a combination of both conditions (*n* = 13;15.6%). Mean cold ischemic time and red blood cell units transfused during LT were 554 (300–941) minutes and 11.4 (0–88) units, respectively. Complications within 30 days were described in 28 (33.7%) patients, mainly bleeding complications in 13 patients, hepatic artery (HA) thrombosis in four and hepatic vein thrombosis in one. Mean follow-up was 76.4 (1–288) months, and during it, four new patients developed thrombotic complications in HA, HA aneurysm, celiac artery, and the portal–splenic–mesenteric vein. HHT relapse in the transplant allograft was detected in 13 (17.1%) patients after 1–19 years (including two fatal recurrences). Overall mortality was 12%. In conclusion, previous assessment of HA anatomy and hyperdynamic circulatory state could reduce LT complications. The risk of relapse in the hepatic graft supports a multidisciplinary follow-up for HHT patients with LT.

## 1. Introduction

Hereditary hemorrhagic telangiectasia (HHT) or Rendu–Osler–Weber syndrome (ORPHA774) is a rare autosomal dominant vascular disease characterized by telangiectases and larger systemic vascular malformations (VMs) [1]. HHT can be diagnosed either through a molecular genetic test or using the Curaçao clinical criteria (recurrent epistaxis, cutaneous/mucosal telangiectasia, visceral VMs, and a first-degree family member with HHT) [1,2]. Mutations in the endoglin (*ENG*) and activin A receptor type II-like 1 (*ACVRL1*) genes are detected in approximately 90% of cases submitted for molecular diagnosis and cause HHT1 and HHT2, respectively [2]. Pathogenetic variants in *ACVRL1* are more frequent in Mediterranean countries versus Northern Europe or North America, where *ENG* mutations predominate [2,3]. Loss of function mutations in *ENG* or *ACVRL1* provoke anomalous vascular overgrowth due to different mechanisms, one of which might be mediated by the overactivation of phosphatidylinositol 3-kinase (PI3K) signaling [2,4,5]. Pulmonary arteriovenous malformations (AVMs) and brain VMs are more common in patients with HHT1, while hepatic VMs are more common in HHT2 [1,2,3]. 

Liver involvement is common in HHT and has been described in up to 70–75% of patients [6,7]. In fact, HHT is the most common cause of congenital liver vascular malformations in adults [8]. Garcia-Tsao described three types of vascular shunting due to the dual blood supply to the liver in HHT patients: arteriovenous (hepatic artery to hepatic vein), arterioportal (hepatic artery to portal vein) and portovenous (portal vein to hepatic vein) [6]. Hepatic-related symptoms are variable and depend on the type of these vascular shuntings: high output cardiac failure (HOCF) and ischemic cholangitis due to arteriovenous shunts; portal hypertension due to arterioportal shunts; and portosystemic encephalopathy related to portovenous shunts [6]. Although symptomatic liver disease represents a minority of cases, some patients develop severe clinical symptoms and require liver transplantation (LT) [9,10,11]. Interestingly, a female predominance is present within the three largest published series of HHT patients with LT, where between 83.3% and 92.8% of patients were women [9,10,11]. Indeed, when assessing gender differences, women have more severe liver involvement among both HHT1 and HHT2 patients [7]. 

Medical treatment with antiangiogenic drugs, such as bevacizumab, or with PI3K or mTOR (mammalian Target of Rapamycin) inhibitors, such as sirolimus, has been described, but with heterogeneous or preliminary results [4,5,12,13,14]. Currently, there is no standardized medical treatment for severe liver disease in HHT to avoid the need for LT. Intriguingly, there is a significant worldwide increase in the number of HHT patients being listed for LT [10,11]. This surgical intervention is more difficult to perform in HHT patients, with higher rates of post-operative complications due to complex and aberrant hepatic vascularization [9]. However, surgical details of LT in HHT patients have been poorly described [9,15,16,17]. More objective data is needed to improve surgical aspects and long-term follow-up of patients requiring LT under the HHT diagnostic umbrella. The aim of the present study is to describe three new HHT patients requiring LT and to perform a systematic review of patients reported to date.

## 2. Materials and Methods

### 2.1. Unpublished New Patients

Consecutive HHT patients were selected from the referral HHT Unit at Hospital Universitari de Bellvitge (Barcelona, Spain). This HHT Unit caters to adult patients from all over Catalonia (Spain), which has about 7.5 million inhabitants, and all patients are included in a database. Those consecutive HHT patients who were referred to our center and who received LT were considered eligible as case reports. Three HHT patients were detected and included. None of these patients had been previously the subject of publications. LT was performed at the Hospital Universitari de Bellvitge in two of the patients and at the Hospital Clínic (Barcelona, Spain) in the third one. Personal and clinical data collection for the study were performed in line with the Spanish Data Protection Act (Ley Orgánica 3/2018 de 5 de diciembre de Protección de Datos Personales). The study was approved by the Clinical Research Ethics Committee of the Hospital Universitari de Bellvitge (Barcelona, Spain; protocol code PR023/22).

### 2.2. Literature Search Strategy

**Figure 1:**
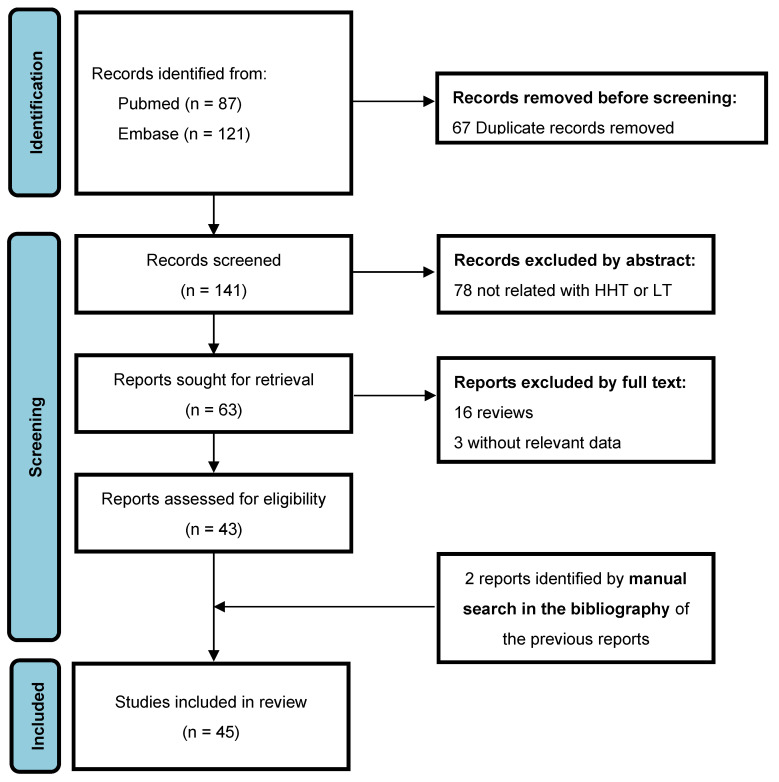
PRISMA flow diagram.

All stages of conception of this systematic review were conducted according to PRISMA guidelines [18]. We performed an unrestricted search of PubMed/MEDLINE and Embase electronic bibliographical databases through February 2022. The search was performed using the terms “hemorrhagic hereditary telangiectasia” or “Rendu-Osler-Weber syndrome” and “liver transplantation” in combination. All published studies describing patients with HHT and LT were selected. Eligible articles were those describing perioperative complications and/or outcomes of HHT patients undergoing LT. Prospective and retrospective cohort studies, patient series, and case reports were included and used for quantitative and qualitative synthesis of data, according to the PRISMA criteria. Reference lists of retrieved articles and review articles were manually searched to extend the original search. Disagreements on study data extraction were resolved by consensus or by discussion. The PRISMA flow diagram relative to the study selection process is provided in Figure 1. 

### 2.3. Outcome Measures

The primary objective was to describe three new HHT patients that required LT and perform a systematic review of the current relevant literature, focusing on perioperative complications within 30 days and long-term follow-up of reported patients.

### 2.4. Data Analysis

Data from included studies were summarized and grouped together to perform quantitative and qualitative analyses as to address the outcome measures. Categorical variables are expressed as frequencies and proportions. Continuous variables are expressed as means with standard deviations (SD) and range. Analyses were performed using IBM SPSS Statistics, version 26.0 for the PC (IBM Corp., Armonk, NY, USA).

## 3. Results

### 3.1. Case Reports

#### 3.1.1. Case 1

A 28-year-old woman with no relevant medical history was diagnosed with HHT meeting all Curaçao criteria. Genetic test detected a pathogenic variant in *ACVRL1*. At diagnosis, an abdominal computed tomography (CT) scan revealed hepatic vascular involvement. Aside from elevated alkaline phosphatase and gamma-glutamyl transferase levels and iron-deficiency anemia related to recurrent epistaxis, the patient initially remained asymptomatic.

Ten years later, she developed right-upper abdominal pain and symptoms suggesting heart failure. Abdominal CT and magnetic resonance imaging (MRI) showed multiple liver AVMs, with hypoperfused areas. A contrast transthoracic echocardiography (TTE) demonstrated a Barzilai’s grade 4 shunt and a cardiac index of 6 L/min/m^2^, with pulmonary hypertension. A thoracic CT confirmed the presence of two pulmonary AVMs. Medical therapy with diuretics and ursodeoxycholic acid was initiated, and both pulmonary AVMs were embolized, reducing shunt grade from 4 to 1. In the following weeks, the patient was readmitted for fever and was ultimately diagnosed with polymicrobial bacteremia (Streptococus anginosus and Haemophilus parainfluenza) related to underlying ischemic cholangitis. With the diagnosis of ischemic cholangitis, LT was indicated. The patient’s Model for End-Stage Liver Disease (MELD) score was 19.

On November 2013, the patient underwent orthotopic liver transplantation (OLT) from a deceased 41-year old donor allograft, with caval preservation and temporary portacaval shunt. The hepatectomy was technically complex, due to multiple AVMs in the hepatic hilum. Multiple diaphragmatic arteries were also found and ligated. Cold ischemic time (CIT) was 620 min, and 16 red blood cells (RBC) units were transfused intraoperatively. The patient’s native liver explants weighed 2640 g. Arterial anastomoses were performed, but immediate hepatic artery thrombosis was observed. Although different arterial revascularization maneuvers were attempted, no arterial flow was achieved. Consequently, the patient was re-listed for emergent re-transplantation, which was performed within 24 hours with a second deceased 40-year old donor allograft. Arterial revascularization was then achieved with a supraceliac aortic anastomosis. Intraoperatively, hepatic artery flow was 220 mL/min. The patient did well post-transplant and was discharged from the Intensive Care Unit (ICU) on postoperative day 3 and from the hospital on postoperative day 14. Nine years after transplantation, she remains alive and well, with normal cardiac index and liver function and a significant reduction in epistaxis severity.

#### 3.1.2. Case 2

A 60-year-old woman with history of arterial hypertension was admitted for congestive heart failure. TTE revealed high a cardiac index of 5.4 L/min/m^2^, with systolic pulmonary hypertension. During admission, the patient developed hepatic encephalopathy. An abdominal CT scan showed multiple hepatic AVMs and portovenous shunts, in addition to significant hypertrophy of the hepatic artery and its branches. 

An extensive evaluation for HHT was carried out. The patient had no family or personal history of epistaxis or telangiectases. A contrast TTE showed a grade 1 right–left shunt, and no pulmonary AVMs were detected on thoracic CT. Cerebral MRI angiography was negative for cerebral AVMs. Genetic test was negative for *ENG*, *ACVRL1*, and *SMAD-4* pathogenic variants. Given the lack of other etiologies for chronic liver disease, a presumptive diagnosis of HHT was made despite the patient only met one Curaçao criteria.

She was admitted for repeated episodes of HOCF and hepatic encephalopathy, which were difficult to manage. Eight months after initial presentation, on November 2007, OLT was performed with a whole deceased 76-year-old donor graft, with caval preservation and temporary portacaval shunt. CIT was 300 min, and no intraoperative transfusions were required. Hepatic artery flow at the end of the procedure was 490 mL/min. The patient was discharged from the ICU on postoperative day 2 and from the hospital on postoperative day 10.

A resolution of HOCF and hepatic encephalopathy were observed during follow-up. However, intrahepatic and extrahepatic bile duct dilation were observed in control imaging tests. The patient had no relevant complications until the eighth post-transplant year, when she was admitted for right-upper abdominal pain and fever. Laboratory tests revealed elevated serum bilirubin, leukocytosis, and slightly prolonged prothrombin time. Abdominal CT was performed and demonstrated intrahepatic bile duct dilation, with the formation of bile lakes suggestive of ischemic cholangitis. In blood cultures, Enterococcus faecium was isolated. An endoscopic retrograde cholangiography (ERC) was performed and was significant for intrahepatic and extrahepatic bile duct dilatation, with one common bile duct stone and sludge. Ten days after admission and 48 hours after ERC, the patient presented sudden hypotension followed by cardiac arrest. Profuse bleeding from the gastrointestinal tract was observed. Following autopsy, the diagnosis of septic shock due to ischemic cholangitis secondary to vascular recurrence of HHT on the transplanted liver was confirmed.

#### 3.1.3. Case 3

The patient was a 55-year-old woman with a history of factor VII deficiency, subclinical hypothyroidism, chronic gastritis, and recurrent epistaxis. Her family medical history was unremarkable. A year prior to presentation, the patient developed mild cholestasis. After an episode of acute cholangitis, cross-sectional imaging was performed and demonstrated numerous AVMs in the liver, located predominantly in the right lobe, with moderate dilation of the intrahepatic bile ducts, though there were no signs of portal hypertension. Though having a negative genetic test for HHT, she was given a presumptive diagnosis of HHT, meeting three Curaçao criteria.

The patient was admitted because of nausea and vomiting and altered liver function in blood tests. Within hours of admission, she developed grade 4 hepatic encephalopathy and required endotracheal intubation and mechanical ventilation. An urgent CT scan was performed, demonstrating the same hepatic AVMs but new-onset hypoperfusion of hepatic segments V, VI, and VIII. A contrast TTE showed no right–left shunt and no pulmonary AVMs were detected on thoracic CT. 

With the diagnosis of ischemic cholangitis secondary to HHT, she was listed for emergency LT. Her MELD score was 21. Twenty-four hours after listing, a liver from a 65-year-old deceased donor was accepted. On August 2020, OLT was performed, with caval preservation and temporary portacaval shunt. The hepatectomy of the native liver was technically complex due to diffuse bleeding from multiple telangiectases located throughout the abdominal cavity, including the parietal peritoneum. The patient´s native liver explant weighed 1200 g. Transplantation of the liver graft was uneventful, and duct-to-duct biliary anastomosis was performed. CIT was 219 min, and five RBC units were transfused intraoperatively.

Immediately postoperatively, the patient presented gastrointestinal bleeding with hemodynamic instability. Upper gastrointestinal endoscopy was performed emergently and demonstrated multiple gastric and duodenal ulcers with clots but no active bleeding. Once the patient stabilized, liver function improved progressively and was discharged from the ICU on postoperative day 10 and from the hospital on postoperative day 31. Currently, she is alive and well, with normal liver function after two years of follow-up.

### 3.2. Included Studies in the Systematic Review

Our search identified 208 potentially eligible studies from Medline and Embase. After exclusion for duplication (*n* = 66), 141 studies were screened. Among them, 78 were excluded because they were not related to LT in HHT and 20 after full text revision for different reasons. We considered three extension follow-up studies (Dumortier 2019 related to Dupuis-Girod 2010; Ullus 2019 related to Saxena 1998; and Sabba 2004 related to Bauer 1995) [16,19,20,21,22,23]. The latter extension study provided an additional case [22]. Two studies were identified by hand-searching in the bibliography of retrieved articles [24,25]. Therefore 45 studies were finally included for analysis [10,11,13,15,16,17,19,20,21,22,23,24,25,26,27,28,29,30,31,32,33,34,35,36,37,38,39,40,41,42,43,44,45,46,47,48,49,50,51,52,53,54,55,56,57]. The PRISMA flow diagram is depicted in Figure 1. 

### 3.3. Patient-Level Main Characteristics of Included Studies

A total of 83 patients who underwent LT for HHT have been included, including the three new cases reported herein (Appendix A Table A1). Globally, 68 (81.9%) patients were female, 12 (14.5%) were male, and three (3.6%) were not described. Mean age (*n* = 79) was 50 (SD 12; 27–72) years. A genetic test was only described from 33 (39.7%) patients, with a disease-causing mutation being found in 30 patients (affecting *ACVRL1* gene in all of them), and with an unidentified pathogenic variant in the remaining three patients. Despite the fact that not all Curaçao criteria were described in all patients, 37 (44.6%) patients met ≥ 3 Curaçao criteria, and were thus classified as having a definite diagnosis. Overall, 57 (68.7%) patients had either a positive genetic test for *ACVRL1* mutations or a definite diagnosis according to Curaçao criteria. 

Most patients suffered from symptomatic heart failure before LT. According to the hepatic vascular shunting classification established by Garcia-Tsao et al., the indications for LT were as follows: predominant HOCF in 40 patients (48.2%), predominant ischemic cholangitis in 19 patients (22.9%), and predominant portal hypertension in four patients (4.8%) [6]. A combination of different clinical presentations was present in 17 patients: HOCF + ischemic cholangitis in 13 patients (15.6%), HOCF + hepatic encephalopathy in three patients (3.6%), and ischemic cholangitis + hepatic encephalopathy in one patient (1.2%). Two patients had cirrhosis/liver failure and in one patient the indication for LT was not described [24]. Values of cardiac output and cardiac index were available from 35 and 30 patients, respectively. All of these patients had a HOCF component, except for four patients that had predominant ischemic cholangitis. Overall, mean cardiac output was 9.68 (SD 2.68; 5.3–18.20), and mean cardiac index was 5.43 (SD 1.29; 3.1–8.1).

Pre-LT related treatments were described in 27 patients: bevacizumab was used in nine patients ([10,11,27,28,29,30,35,37,57]; all studies were published after 2015), hepatic artery (HA) embolization/ligation/dearterialization was performed in 11 patients ([20,22,35,42,47,51,56]; nine of them were published before 2010), and pulmonary AVM embolization was described in only two patients (Case 1, [46]). Remarkably, cholecystectomy was performed in nine patients before LT (all of them were published before 2010) and eight of them had ischemic cholangitis as an indication for LT. Four pregnant women requiring LT due to ischemic cholangitis were described at 33, 29, and 13 weeks of gestation (unknown in one patient) [25,45,52,54].

### 3.4. Patient-Level Transplant Surgery Characteristics and Perioperative Complications of Included Studies

MELD score was infrequently described (*n* = 10 patients), and its mean value was 11.5 (SD 6; 6–22). Mean CIT (*n* = 31 patients) and mean RBC units (*n* = 35 patients) during LT surgery were 554 minutes (SD 130; 300–941) and 11.4 units (SD 17; 0–88), respectively. Surgical technique was described in 45 patients, and information provided was mostly related to the types of anastomoses performed. Type of graft used was poorly described: deceased whole graft in 13 patients, deceased split in one, and living donor in two patients. LT was described as elective in 10 patients and urgent in six (not described in the rest of patients). Different surgical techniques were used. Older studies mostly referred the classical technique, with caval replacement performed with or without venovenous bypass, whereas more recent studies mostly referred the caval preservation (piggyback) technique with anastomosis with two or three hepatic veins (with or without cavotomy) or a modified piggyback technique (including end-to-side or side-to-side cavocaval anastomosis). 

Perioperative complications within 30 days were described in 28 (33.7%) patients (Appendix A Table A2). Bleeding complications occurred in 13 (15.6%) patients: gastrointestinal bleeding in three patients (one died on day 11), intracerebral bleeding in two (one died on day 2), hematoma in Morrison space in one patient, massive pulmonary bleeding in two patients (both died) and no specified in five patients (Case 3, [12,17,21,24,26,33,39,44,49,56]). Importantly, these last two patients with pulmonary bleeding were not screened for pulmonary AVM prior to LT [24,49]. HA thrombosis was described in four patients, hepatic vein (HV) thrombosis in another and splenic rupture were reported in another two patients (Case 1, [11,12,47,56]). Mortality within 30 days occurred in seven (8,4%) patients and was due to intracardiac thrombus (after 7.1 h), intracerebral bleeding (on day 2), massive pulmonary bleeding in two patients (on days 6 and 7), massive gastric bleeding (on day 11), and heart failure in two patients (both on day 30) [12,17,24,36,45,49]. 

### 3.5. Patient-Level Long-Term Follow-Up of Included Studies

Long-term data was available from 69 out of the 76 discharged patients, and mean follow-up was 76.4 (SD 72; 1–288) months (Appendix A Table A2). Thrombotic complications were described in four new patients: HA thrombosis, HA aneurysm thrombosis, celiac artery thrombosis, and portal–splenic–mesenteric venous thrombosis [10,15,47]. Regarding the four pregnant women that needed LT, one died due to intracardiac thrombus at 7.1 hours after LT, two were alive at 12 and 65 months, and no follow-up data is available for the fourth [25,45,52,54]. Interestingly, HHT relapse in the transplanted allograft was detected in 13 (17.1%) among 76 discharged patients between 1 and 19 years post-transplant, including two patients that died due to recurrence complications (Case 2, [12,19,20,21,22,23,42]). Three patients died during follow-up due to myocardial infarction (at day 34), ischemic cholangitis due to recurrence of hepatic VM (8 years later), and portal hypertension due to recurrence of hepatic VM (6 years later) (Case 2, [32,42]). Thus, overall mortality was 12%. 

## 4. Discussion

The first case of LT for HHT reported in the English literature was published by Bauer et al. in 1995 (23). Since then, 83 patients are reported herein, with an 88% of survival after a mean follow-up of more than six years. According to the classification established by Garcia-Tsao et al, the main indication for LT in our series was HOCF (isolated or in combination), as it occurred in 56 (67.5%) reported patients, followed by ischemic cholangitis in 33 (39.7%) patients [6]. These different subtypes of hepatic vascular shunting usually coexist but one of them predominates functionally and, at the same time, show fluctuation and transition from one clinical picture to another [58]. With the exception of ischemic cholangitis as an urgent indication to LT, there is no general consensus on the best timing in considering transplantation for HOCF. MELD score was designed for cirrhotic patients, and HHT patients usually need to be granted a MELD exception to be included and prioritized in the LT waitlist [59,60]. 

Invasive treatments performed prior to LT, such as ligation, banding, or embolization of the HA, are not recommended because of high associated morbidity and mortality [61]. In a previous revision by Whiting et al., 10 (41.7%) out of 24 patients who underwent HA embolization experienced significant morbidity (two required emergency LT and four experienced serious complications) and mortality (four patients) [62]. It is important to note that these techniques were used in the oldest reports of our systematic review, so complications and survival reported herein might be disproportionately influenced by the use of these treatments before LT [9,63,64]. Moreover, transjugular intrahepatic portosystemic shunt (TIPS) in HHT has been described in an attempt to treat ascites and variceal hemorrhage, though its application in this context is largely unsuccessful [42,65]. As such, LT is currently the recommended surgical option for HHT patients with severe hepatic vascular involvement [61]. Bevacizumab has shown to improve cardiac index in HHT patients with HOCF and may potentially alleviate the need for LT in HHT patients with HOCF in some case reports [12,61,66]. However, its use as a bridge therapy for LT, as it was used in nine of herein reported patients, is questionable, because of unpredictable efficacy (considering the strength of the response, time to improvement, and duration of the benefit), toxicity (such as gastrointestinal perforation, thromboembolic events, hypertensive crisis, or nephrotic syndrome) and surgical complications with respect to angiogenesis-dependent phenomena such as wound repair or healing of anastomoses [10,11,27,28,29,30,35,37,57,67].

Reported cases reflect the complexity of LT for HHT, with high blood transfusion requirements and prolonged graft CIT. Although reports are heterogeneous and surgical complications are not always described, perioperative complications are high compared to other indications [68]. The HA in HHT patients may be dilated and/or tortuous and should be handled with care. At the start of hilar dissection during recipient hepatectomy, it may be advantageous to place a bulldog or other vascular clamp proximally on the common hepatic artery before dissecting and dividing branches distally, in order to avoid propagation and potentially catastrophic consequences of any intimal dissection. Moreover, this maneuver also contributes to reducing the hyperdynamic state and potential risk for bleeding. Arterial anastomosis should be performed using a recipient artery of adequate caliber and wall consistency; in fact, severely dilated or aneurysmatic recipient artery should be avoided. Adequate flow through the anastomosis should be ideally confirmed intraoperatively using ultrasound flow measurement confirming flow rate and triphasic waveform. Additionally, arterial telangiectases may play a role in arterial steal, which may help explain the high incidence of HA thrombosis after LT in HHT patients. HA thrombosis was described in six out of the 83 reported patients (four in the in-hospital setting after LT and two during follow-up) and resulted in re-transplantation in two (Case 1, [15]). Moreover, it is important to note that fatal pulmonary bleeding was described in two patients, so, HHT patients should also be screened for pulmonary AVM and, if present, embolization should be considered previously to LT [24,49].

Intrahepatic relapse of the hallmark lesions of HHT (hepatic telangiectasia or large AVMs) has been described in 13 patients after LT (Case 2, [19,20,22,23,42]). In a recent study by Dumortier et al., which included long-term follow-up of 14 patients (this series include one new female patient from the series published by Dupuis-Girod), HHT recurrence was diagnosed by abnormal radiological features in 8 (seven female) and confirmed in three out of the five patients with liver biopsy. The median interval between LT and recurrence diagnosis was 127 months (range: 74–184), increasing the risk of recurrence over time with an estimated cumulative risk of 0% at 5 years, 16.7% at 10 years, 47.9% at 15 years, and 87% at 20 years [16,19]. Four previous cases of HHT recurrence were reported and diagnosis of HHT recurrence was made at 5, 8, 10, and 19 years after LT [20,22,23,42]. In our case 2, allograft recurrence was detected in the context of symptomatic ischemic cholangitis eight years after LT. This patient, together with one described by Cura et al., who developed portal hypertension five years after LT, are together the only two reported symptomatic relapses after LT (both patients died, one and 11 months after the diagnosis of HHT recurrence) (Case 2, [42]). All these findings suggest that HHT recurrence is a very late event after LT, and even may be underdiagnosed. Thus, these patients require life-long follow-up in the setting of a multidisciplinary HHT Unit. 

The pathophysiology of LT recurrence is still unclear. However, Dumortier et al. found the coexistence of an angiogenic process combined with endothelial microchimerism, as shown by the presence of vascular lining cells of recipient origin in three female recipients of male liver grafts [19]. Microchimerism is a well-known feature after LT, but it may develop into an aberrant angiogenic process due to abnormal endothelial cells repopulating the liver graft [69,70]. The use of an mTOR inhibitor-based immunosuppression regimen may be useful for these patients, as sirolimus block the overactivated PI3K signaling pathway in HHT reducing vascular growth and may reduce allograft recurrence [4,5,13,14,71].

In conclusion, the management of LT in HHT patients is complex. An optimal screening and management of VMs prior to LT is needed. Surgical challenges should include the assessment of HA anatomy and the hyperdynamic circulatory state to reduce surgical complications. The risk of relapse in the hepatic graft supports the need for a multidisciplinary follow-up of these patients.

## Data Availability

Not applicable.

## Appendix A

**Table A1:** Main characteristics of the three new cases and all included patients.

Study, Year (Ref)	*n* (Sex)	Age (Years)	Gene	CC	Transplant Indication/s	Pretransplant Clinical Characteristics	CO (L/min)/CI (L/min/m^2^)	Pre-LT Related Treatments
Case 1	1 (F)	33	ACVRL1	4	HOCF + Ischemic cholangitis	Right-upper abdominal pain, polymicrobial bacteriemia.	-	PAVMs embolization.
Case 2	1 (F)	60	Negative	1	HOCF + Hepatic encephalopathy	Heart failure, hepatic encephalopathy	-	-
Case 3	1 (F)	55	Negative	2	Hepatic encephalopathy + Ischemic cholangitis	Grade 4 hepatic encephalopathy	-	-
Perrodin, 2022 [57]	1 (F)	72	Negative	2 (E, V)	Ischemic cholangitis	Pulmonary hypertension with dyspnea NYHA III, recurrent ischemic cholangitis episodes with multiple abscesses		BVZ (6 doses) Sildenafil
Olsen, 2020 [26]	1 (F)	65	ACVRL1	2 * (E, V)	HOCF + Ischemic cholangitis	Dyspnea NYHA II, peripheral edema and AF. PAVMs previously embolized.	-/6.6	-
Morales, 2020 [27]	1 (F)	63	-	2 * (E, V)	HOCF	Dyspnea NYHA III-IV, PHTN, AF and recurrent epistaxis and episodes of upper GI bleeding.	5.9/-	BVZ Sildenafil
Vazquez, 2020 [28]	2 (F/M)	36/45	-	2 * (T, V)	HOCF	P1: Dyspnea NYHA III-IV. P2: Dyspnea NYHA III-IV. Many previous cardiac comorbidities (prosthetic aortic valve, tricuspid valve plasty, closure of a congenital interventricular communication, AF).	P1: 9.3/6.2 P2: 8/4.1	Both received BVZ.
Iyer, 2019 [11]	5 (all F)	40/57/57/50/69	-	P1: 2 * (V, FH) P2: 3 * (E, V, FH) P3: 3 * (E, V, FH) P4: 3 * (E, V, FH) P5: 3 * (E, V, FH)	P1: ischemic cholangitis + HOCF P2,3,4 and 5: HOCF	P1: Hepatic abscesses and MSSA bacteremia + arrhythmia. P2: Shortness of breath and volume overload signs. P3, P4 & P5: Shortness of breath and volume overload signs + PHTN	P1: 10.2/6 P2: 8.6/4.7 P3: 9.1/4.1 P4: 5.3/5.7 P5: 8.5/4.3	Some received BVZ (not identified)
Dumortier, 2019 [29]	1 (F)	60	-	2 * (V, FH)	HOCF + Hepatic encephalopathy	Dyspnea, fluctuating confusion and somnolence.	-/4.9	BVZ
Álamo, 2019 [30]	1 (F)	62	-	1 * (V)	HOCF	Severe heart failure	7.1/-	BVZ Sildenafil
Ejiri, 2019 [31]	1 (F)	52	-	4	HOCF and PHTN secondary to hepatic AVMs	WHO class IV (dyspnea at rest)	9/-	-
Ionescu, 2018 [32]	1 (M)	59		2 * (E, V)	HOCF	MEN-I, transitory ischemic attack, and AF.	15.3/7.6	-
Barajas, 2018 [33]	1 (F)	40	ACVRL1	3 * (E, T, V)	HOCF + Hepatic encephalopathy	Numerous admissions due to HOCF, respiratory infections and hepatic encephalopathy	12/-	-
Ahumada, 2017 [34]	1 (M)	51	-	2 * (T i V)	HOCF	Dyspnea NYHA III.	7.1/-	HAE (5 procedures)
Chavan, 2017 [35]	1 (F)	-	-	-	HOCF	Dyspnea NYHA III	-	Repeated doses of BVZ without improvement, receiving HAE
Felli, 2017 [10]	1 (F)	66	-	3 * (E, T, V)	HCOF	Cardiac cirrhosis	-	BVZ
Lecler, 2015 [36]	1 (F)	66	-	3 * (E, T, V)	HOCF + ischemic cholangitis	Jaundice and painful hepatomegaly. Biliomas.	11/6.9	-
Maestraggi, 2015 [37]	1 (F)	63	ACVRL1	4	Ischemic cholangitis	Right-upper abdominal pain. Biliomas. Bilateral pulmonary embolisms, thrombosis in the right atrial cavity and thrombosis of the right hepatic vein.	-	BVZ
Maggi, 2013 [15]	2 (all F)	44/64	-	4	HOCF	-	P1: 5.6/- P2: 10/-	-
Chawala, 2011 [38]	1 (F)	44	-	3 * (E, T, V)	HOCF	Palpitations, dyspnea, and hematochezia	9.1/5.8	-
Cag, 2011 [39]	4 (M/M/F/M)	60/65/40/64	-/ACVRL1/-/-	P1: 3 (E, T, V) P2: 3 (T, V, FH) P3: 4 P4: 2 (T, V)	P1: VHB induced cirrhosis P2, 3 & 4: HOCF	P1: HBV-induced cirrhosis + 2 previous renal transplantation P2: - P3: - P4: HBV-induced cirrhosis	P1: -/- P2: 10.5/- P3: 7.9/4.9 P4: 7.8/4.1	No HAE. Medical treatments had been used (not specified).
Buscarini, 2011 [40]	2 (-/-)	-/-	-	All: at least 2 *	P1: HOCF P2: Portal hypertension	P1: Dyspnea NYHA class III–IV P2: Refractory ascites	P1: -/6.2 P2: -/-	-
Dupuis-Girod, 2010 [12] (and Dumortier, 2019 [19])	13 (12F, 1M)	36/50/38/43/55/65/57/63/65/58/46/61/33	ACVRL1 (all 13)	All: 2 * (E, V; except for 1 patient with unknown E)	HOCF: 9 patients Ischemic cholangitis: 3 patients Both: 1 patient	For the 9 patients with HOCF: dyspnea NYHA II-IV; 1 patient with severe PHTN For the 3 patients with ischemic cholangitis: right upper abdominal pain For the 1 patient with HOCF/ischemic cholangitis: dyspnea NYHA IV and right upper abdominal pain	Overall, mean CO 8.8 (5.3–14.1). CI values were: P1: -/5.8 P2: -/6.1 P3: -/5.5 P4: -/3.1 P5: -/6.8 P6: -/5.1 P7: -/5.6 P8: -/3.3 P9: -/3.5 P10: -/4.8 P11: -/4.0 P12: -/8.1 P13: unknown	-
Brenard, 2010 [41]	3 (all F)	29/53/32	- ACVRL1 ACVRL1	All: at least *	P1: Ischemic cholangitis P2: HOCF P3: Ischemic cholangitis	-	P1: -/- P2: 12.41/- P3: -/-	-
Cura, 2010 [42]	1 (M)	50	-	2 * (V, FH)	Portal hypertension	Recurrent episodes of haemobilia and GI variceal bleeding. Cirrhosis.	-	Various HAEs. Left hepatic lobectomy and cholecystectomy
Lee, 2010 [43]	1 (M)	47	ACVRL1	1	Ischemic cholangitis	Dyspnea on exertion, epigastric pain, jaundice, and fevers.	18.2/-	-
Nuñez Viejo, 2010 [44]	1 (F)	48	-	3 * (E, T, V)	HOCF	NYHA IV	10.6/-	-
Mavrakis, 2009 [45]	1 (F)	32	-	3 (E, V, FH)	Fulminant hepatic failure and septic shock	At 33 weeks gestation, weight loss, upper quadrant abdominal pain, nausea, and intermittent vomiting.	-	Vaginal delivery. Elective laparoscopic cholecystectomy
Skaro, 2006 [13]	1 (F)	53	-	3 * (T, V, FH)	HOCF	Jaundice, cachexia, and ascites. Chronic refractory anemia despite multiple blood transfusions.	-	None described
Domínguez, 2005 [46]	1 (F)	32	-	2 (V, FH)	Ischemic cholangitis	Right upper quadrant abdominal pain, hyperthermia and jaundice.	-	Laparoscopic cholecystectomy. PAVM embolized.
Thevenot, 2005 [47]	2 (all F)	59/62	ACVRL1 (both)	P1: 3 * (E, T, V) P2: 4	P1: HOCF + Ischemic cholangitis after HAE P2: HOCF	P1: fever, jaundice, cardiac failure with dilated jugular veins, bilateral pleural effusion, peripheral edema. P2: breathing difficulties.	P1: 8.4/5.1 P2: 10.7/6.6	P1: Left HAE. P2: None
Argyriu, 2005 [48]	2 (F/M)	36/60	ACVRL1 (both)	P1: 3 (E, T, V) P2: 4	HOCF	P1: Increased right heart load, dyspnea. P2: Increased right heart load, arrhythmia, dyspnea, ascites, and esophageal varices.	-	-
Sabbà, 2004 [22]	1 (F)	49	-	2 * (V, FH)	HOCF + Biliary sepsis after hepatic artery ligation.	-	-	HA ligation
Giacomoni, 2004 [24]	1 (-)	-	-	1 * (V)	-	-	-	-
Aseni, 2003 [49]	1 (M)	29	-	3 (E, T, V)	Hepatopulmonary syndrome	Severe respiratory distress	-	-
Blewitt, 2003 [50]	1 (F)	34	-	2 * (V, FH)	Ischemic cholangitis	Continuous right upper quadrant abdominal pain	-	Laparoscopic cholecystectomy.
Azoulay, 2002 [17]	6 (F/F/F/F/M/F)	38/41/49/38/67/48	-	P1: 3 (E, V, FH) P2: 4 P3: 4 P4: 2 (V, FH) P5: 4 P6: 3 (T, V, FH)	P1, 3 and 4: Ischemic cholangitis P2: Portal hypertension P5: Portal hypertension P6: HOCF + cholangitis	P1: Repeated biliary sepsis and abscess drainages P2: Ascites, esophagi varices, hypertensive gastritis. Abscess drainage. P3: -. P4: Esophagi varices, hypertensive gastritis. Abscess drainage P5: Ascites and esophagi varices, hypertensive gastritis. PHTN. P6: -	-	No HA ligation or HAE. P5: TIPS
Pfitzmann, 2001 [51]	4 (all F)	45/69/54/55	ACVRL1 (All)	P1: 2 * P2: 2 * P3: 2 * P4: 2 * All (T, V)	P1, 4: HOCF + ischemic cholangitis P2, 3: HOCF	P1: NYHA III-IV, abdominal pain, weight loss and icterus. P2: NYHA III-IV, GI bleeding, ascites, recurrent pulmonary embolisms, Ventricular arrhythmias. P3: NYHA III-IV, ascites, edema, PHTN, tricuspid insufficiency grade IV. P4: NYHA III-IV, pleural effusions and ventricular arrhythmias.	P1: 8.8/- P2: 13.3/- P3: 12/- P4: 8/-	P1: HAE. P2–3: - P4: 6 HAE and surgical HA banding
Hillert, 2001 [52]	1 (F)	39	-	4	Ischemic cholangitis	At 29 weeks gestation, diffuse abdominal pain and several episodes of GI bleeding. Billroth I resection of the stomach. Hepatic vein thrombosis.	-	Cesarean Delivery Right HAE
Le Corre, 2000 [53]	1 (F)	40	-	3 * (E, V, FH)	HOCF	Previous right pulmonary lobectomy. NYHA II-III.	12.5/7.35	-
Boillot, 1999 [54]	3 (all F)	36/50/42	-	P1: 3 (E, V, FH) P2: 3 *(E, V, FH) P3: 2 * (E, V)	P1: HOCF, Ischemic cholangitis P2 and 3: HOCF	P1: At 13 weeks gestation, right-upper abdominal pain. Cardiac failure associated with PHTN. Multiple Hepatic abscess P2: Refractory ascites and PHTN P3: Acute right-sided HF and massive edema and ascites.	P1: 9.1/- P2: 11.3/- P3: 10.8/-	P1: cesarean delivery. cholecystectomy. P2 and P3: -
Neuman, 1998 [55]	1 (F)	45	ACVRL1	1 * (V)	HOCF + Ischemic cholangitis	Previous partial left pneumonectomy for PAVMs. Esophageal and gastric varices grade IV	8.8/-	Complete liver dearterialization. Cholecystectomy
Odorico, 1998 [56]	2 (all F)	48/47	-	2 (E, V)	P1: Ischemic cholangitis P2: Ischemic cholangitis	P1: Large bilomas, multiple hepatic abscesses and polymicrobial bacteriemia. Hepatic encephalopathy after embolization P2: Abdominal pain, jaundice, fever. Multiple bilomas. Polymicrobial bacteriemia (including *Candida Albicans*). Encephalopathy after embolization	P1: 6.6/-	P1: cholecystectomy.Embolization of pancreaticoduodenal arteries P2: HAE. Open cholecystectomy
Saxena, 1998 [21] (and Ullus 2019 [20])	1 (F)	43	-	3 * (T, V, FH)	Ischemic cholangitis	Ascites, pleural effusion, wasting, and extreme fatigue.	-	Previous surgical dearterialization of the HA.
Mclnroy, 1998 [25]	1 (F)	31	-	1 (V) *	Ischemic cholangitis	Gravida. Right upper quadrant pain, low-grade fevers, and elevated liver enzyme levels. Hematemesis. Confirmed bacteriemia.	-	Vaginal delivery. Open cholecystectomy
Bauer, 1995 [23] (and Sabbà 2004 [22])	1 (F)	33	-	4	HOCF + Ischemic cholangitis	Severe upper abdominal pain, sepsis with isolation of *Streptococcus faecalis* due to ischemic cholangitis	-	-

**Abbreviations**: AF: atrial fibrillation; BVZ: bevacizumab; CC: Curaçao Criteria; CI: cardiac index; CO: cardiac output; E: epistaxis; F: female; FH: family history; GI: gastrointestinal; HA: hepatic artery; HAE: hepatic artery embolization; HOCF: high output cardiac failure; M: male; NYHA: New York Heart Association Functional Classification; PAVMs: pulmonary arteriovenous malformations; PH: portal hypertension; PHTN: pulmonary hypertension; T: telangiectases; V: visceral involvement. (*) not all Curaçao Criteria described.

**Table A2:** Transplant surgery characteristics and outcomes of the three new cases and all included patients.

Study, Year (Ref)	*n*	MELD	CIT (min)	RBC (U)	Surgical Technique	Perioperative Complications within 30 Days	Long Term Follow-Up
Case 1	1	19	620	16	Caval preservation and temporary portocaval shunt, side-to-side caval anastomosis	HA thrombosis Urgent re-transplantation	8 years, alive
Case 2	1	11	300	0	Caval preservation and temporary portocaval shunt	-	Death at 8 years due to ischemic cholangitis and recurrence of HHT in transplanted liver
Case 3	1	21	219	5	Caval preservation and temporary portocaval shunt	Diffuse intraoperative bleeding from peritoneal telangiectasias Post-operative GI hemorrhage	18 months, alive
Perrodin, 2022 [57]	1	-	300	-	Side-to-side cavo-caval anastomosis (piggy-back technique) and an end-to-end portal anastomosis. Arterial reconstruction proved challenging, with abnormally fragile recipient arteries of large diameter, leading to a significant donor-recipient size mismatch.	The postoperative course was uneventful.	36 months, alive
Olsen, 2020 [26]	1	-	-	-	-	Intraabdominal hemorrhage from phrenic artery requiring surgical reintervention Cholangitis	9 months, alive
Morales, 2020 [27])	1	15	-	0	-	None described	-
Vazquez, 2020 [28]	2	-	-	-	-	-	P1: 1 year, alive P2: 2 years, alive
Iyer, 2019 [11]	5	P1: 8 P2: 6 P3: 11 P4: 8 P5: 6	-	-	-	P1: HA thrombosis P2: - P3: HA thrombosis and acute rejection P4: HV thrombosis, requiring surgical thrombectomy and liver resection P5: -	P1: 104 months, alive P2: 83 months, alive P3: 88 months, alive P4: 100 months, alive P5: 87 months, alive; HA aneurysm diagnosed 1 y post-LT
Dumortier, 2019 [29]	1	-	-	-	-	-	30 months, alive
Álamo, 2019 [30]	1	-	-	-	Arterial anastomosis with recipient’s left HA	-	-
Ejiri, 2019 [31]	1	-	-	-	-	None described.	Alive, follow-up unknown
Ionescu, 2018 [32]	1				End-to-side arterial anastomosis	Significant hypoxemia with normal pulmonary arteriography, resolved with prone ventilation	Death at day 34 after LT due to acute MI
Barajas, 2018 [33]	1	-	-	-		Intraoperative hemorrhage, signs of portal hypertension	-
Ahumada, 2017 [34]	1	12	711	4	Modified piggyback technique, with hepaticojejunostomy for biliary reconstruction	None	9 years, alive
Chavan, 2017 [35]	1	-	-	-	-	-	-
Felli, 2017 [10]	1	-	600	-	Caval preservation, duct-to-duct biliary anastomosis	-	14 months, alive
Lecler, 2015 [36]	1	-	-	-	-	Death due to heart failure after first post-operative month	-
Maestraggi, 2015 [37]	1	-	-	-	-	-	1 year, alive
Maggi, 2013 [15]	2	P1: 17 P2: 6	-	2 2	P1: side-to-side caval anastomosis, SMA jump graft interposed between donor RHA and recipient GDA P2: side-to-side caval anastomosis, arterial anastomosis performed with recipient splenic artery	-	P1: 3 years, alive (but re-transplantation at 2 months for HA thrombosis) P2: 1 year, alive
Chawala, 2011 [38]	1	-	510	1	-	-	5 months, alive
Cag, 2011 [39]	4	-	380 535 941 755	6 6 0 6	Modified piggyback technique	P1: - P2: - P3: ongoing postreperfusion coagulopathy, hemofiltration required P4: GI hemorrhage from small bowel angiomas, requiring transfusion	P1: 9 years, alive P2: 7 years, alive P3: 6 years, alive P4: 3 years, alive
Buscarini, 2011 [40]	2	-	-	-	-	-	P1: 14 months, alive P2: 56 months, alive
Dupuis-Girod, 2010 [12] (and Dumortier, 2019 [19])	13		Median 530 min (80–825)	Mean: 6 (0–26)	Caval preservation in 4 cases, VVB in one case Enlarged HA in all cases, with HA aneurysm observed in 3	HA thrombosis (*n* = 1), hemorrhage requiring surgical reintervention (*n* = 2), biliary fistula (*n* = 2), biliary stenosis (*n* = 1), incisional hernia (*n* = 1), death due to cardiac failure at 1 month (*n* = 1)	P1: 16 years, alive P2: 15 years, alive. At 5 years, developed partial thrombosis of the aneurysmal dilatation of the HA that was left uncorrected at the time of OLT. Aneurysmal dilatation was resected and arterial flow to the graft was successfully restored. At 8.5 years, developed an acute cerebral hemorrhage (attributable to an AVM). P3: 14 years, alive P4: 11 years, alive. At 10 years, a computed tomography revealed hepatic hypervascularization; a liver biopsy was compatible with peliosis. P5: 10 years, alive P7: 8 years, alive P8: 7 years, alive P9: 6 years, alive P10: 6 years, alive P11: 4 years, alive P12: 4 years, alive P13: 4 years, alive
Brenard, 2010 [41]	3	-	-	-	-	-	P1: 1 year, alive P2: 3 months, alive P3: 3 years, alive
Cura, 2010 [42]	1	-	-	-	Roux-en-Y choledocho-jejunostomy and aorto-hepatic bypass graft.	-	At 5 years, the patient presented PH secondary to the development of fistulae in the transplanted graft. A TIPS was created, and a percutaneous liver biopsy showed recurrent vascular proliferation. The patient died 11 months later due to polymicrobial bacteremia secondary to colitis with esophageal varices, ascites and hydrothorax
Lee, 2010 [43]	1	22	-	-	-	-	-
Nuñez Viejo, 2010 [44]	1	-	-	-	-	Hematoma in Morrison space, a respiratory infection, diarrhea, kidney function impairment and thrombocytopenia.	5 years, alive
Mavrakis, 2009 [45]	1	-	-	-	-	After donor liver implantation, the patient developed an intracardiac thrombus and a subsequent cardiac arrest. All attempts at resuscitation failed and the patient died after 7.1 h of total operative time.	-
Skaro, 2006 [13]	1	-	-	-	-	-	1 year, alive
Domínguez, 2005 [46]	1	-	-	-	-	-	3 months, alive
Thevenot, 2005 [47])	2	-	510 390	25 5	-	P1: PV clamping resulted in splenic rupture, first requiring a temporary portocaval shunt and thereafter splenectomy. P2: -	P1: at 4 months developed celiac artery thrombosis followed by resumed permeability under fluindione. 3 years later, alive. P2: at 1 year developed mesenteric and splenic vein thrombosis extending to the portal trunk, solved after 1 year anticoagulation. Two years later, alive.
Argyriu, 2005 [48]	2	-	-	-	-	-	P1: 4 years, alive P2: 1 year, alive
Sabbà, 2004 [22]	1	-	-	-		-	P2: 8 years, alive; relapse on computed tomography scan
Giacomoni, 2004 [24]	1	-	-	-	Hepatic right lobe living related transplant. The right hepatic duct was split before the parenchymal transection with a fully perfused liver, after dissection of the right HA, the right PV, and the right HV. VVB was not used.	Died on day 7 due to massive pulmonary bleeding, because of the rupture of pulmonary AVM.	-
Aseni, 2003 [49]	1	-	-	-	-	On day 3, bilateral pulmonary atelectasis and pulmonary edema was detected. A bronchoscopy revealed blood clots in the left main bronchus. On day 6, massive hemoptysis developed, and the patient died.	-
Blewitt, 2003 [50]	1	-	-	-	-	Complicated post-operative course (not explained).	3 years, alive
Azoulay, 2002 [17]	6	-	680 578 773 457 649 612	16 18 61 4 88 77	Difficult dissection in all patients due to the hypertrophy and collateral arterial network aggravated by prior procedures and/or severe PH. Extracorporeal VVB was performed in all patients. Pericardium had to opened in P4 to control the suprahepatic vein. In all 6 patients the retrohepatic vena cava was resected for whole-liver transplantation.	P1–4: - P5: severe encapsulating peritonitis prior to surgery and required 88 RBC units during surgery. Died on day 2 due to massive hemorrhage in the cerebral ventricles with no visible vascular anomaly. P6: a hemostatic gastrostomy had to be performed during the procedure due to the rupture of a gastric AVM. Died on day 11 due to rapid (2 h) fatal massive gastric bleeding.	P1–4: 3 to 7.5 years (median 4 years and 9 months), alive
Pfitzmann, 2001 [51]	4	-	-	-	LT was performed using standard surgical techniques and VVB. After completion of all vascular anastomosis with end-to-end cavo-caval, porto-portal, and arterio-arterial anastomosis biliary anastomosis was performed as side-to-side choledocho-choledochostomy.	P1, 2 and 4: - P3: anuria for 2 weeks and a pneumonia	48–68 months, alive
Hillert, 2001 [52]	1	-	-	-	During LT, a prophylactic sternotomy was performed at the time of clamping of the IVC to obtain optimal access to the intracardial thrombus and prevent pulmonary embolism.	-	1 year, alive
Le Corre, 2000 [53]	1	-	-	3	Surgical procedure consisted of preservation of the IVC, side-to-side caval anastomosis with the use of TPCS. Neither clamping of the IVC nor VVB were needed.	-	-
Boillot, 1999 [54]	3	-	-	26 7 9	P1: a complete vascular exclusion of the liver was performed before hepatic mobilization to avoid a septic embolisms. Therefore, extracorporeal VVB was necessary. Because of the enlarged liver, the upper vascular hepatic exclusion was achieved by clamping the IVC in its intrapericardial region. P2 and P3: extracorporeal circulation was not used; nevertheless, in P3 a piggyback procedure was performed because of the discrepancy between the donor and the recipient IVC. In all patients, the HA was tied and cut before liver dissection to minimize the risk of hemorrhage caused by telangiectases and to obtain an early reduction in central venous pressure and cardiac output during surgery. Because of the enlarged caliber of the patient’s HA, the arterial anastomosis was made between the recipient’s HA and the celiac patch of the donor artery.	P1: complicated vascular and hemodynamic alterations with subsequent greater blood loss. P2 and 3: none described.	P1: 65 months, alive P2: 53 months, alive P3: 29 months, alive
Neuman, 1998 [55]	1	-	-	-	-	-	-
Odorico, 1998 [56]	2	-	-	-	P1: A suprahepatic cava–to–HV anastomosis using the piggyback technique was performed without the use of VVB. A standard donor HA–to–recipient HA anastomosis was performed. Choledochoscopic extraction of common bile duct stones/sludge in the recipient duct was necessary before performing a biliary anastomosis over a T-tube. P2: The donor liver was revascularized using a piggyback caval anastomosis technique. A friable recipient PV required ligation and vascular reconstruction with a conduit of donor iliac vein from the recipient SMV to the donor PV.	P1: PV clamping resulted in splenic rupture, requiring spleneomy P2: incomplete fascial closure secondary to bowel edema. Reintubation for transient ventilatory failure; hemorrhagic cerebrovascular accident involving the right frontoparietal motor cortex; *Staphylococcus* central catheter sepsis; *Enterococcus faecalis* wound infection; PV thrombosis.	P1: 1 year, alive P2: 9 months, alive
Saxena, 1998 [21] (and Ullus 2019 [20])	1	-	-	-	-	Severe bleeding during surgery	19 years, alive. Relapse (AVMs at hepatic MRI); aneurysm of the right HA treated with coil embolization.
Mclnroy, 1998 [25]	1	-	-	-	-	-	-
Bauer, 1995 [23] (and Sabbà 2004 [22])	1	-	-	-	-	-	24 months, alive P1: 10 years, alive; relapse on celiac and mesenteric arteriography

**Abbreviations**: AVM: arteriovenous malformation; CIT: cold ischemic time; GDA: gastroduodenal artery; GI: gastrointestinal; HA: hepatic artery; HV: hepatic vein; IVC: inferior vena cava; LT: liver transplant; MELD: Model for End-Stage Liver Disease score; MI: myocardial infarction; PH: portal hypertension; PV: portal vein; RBC: red blood cell units; RHA: right hepatic artery; SA: splenic artery; SMA: superior mesenteric artery; SMV: superior mesenteric vein; TPCS: temporary portocaval shunt; U: urgent; VVB: venovenous bypass.
